# The influence of anemia on one-year exacerbation rate of patients with COPD-PH

**DOI:** 10.1186/s12890-018-0693-6

**Published:** 2018-08-23

**Authors:** Wei Xiong, Mei Xu, Bigyan Pudasaini, Xuejun Guo, Jinming Liu

**Affiliations:** 1 Department of Respiratory Medicine, Xinhua Hospital, Shanghai Jiaotong University School of Medicine, No. 1665, Kongjiang Road, Yangpu District, Shanghai, 200092 People’s Republic of China; 2 Department of Cardiopulmonary Circulation, Shanghai Pulmonary Hospital, Tongji University School of Medicine, Shanghai, China; 3 Department of Pediatrics, Dinghai Community Health Service Center, Tongji University School of Medicine, Shanghai, China;Department of Pediatrics, Kongjiang Hospital, Yangpu District, Shanghai, China

**Keywords:** COPD, Pulmonary hypertension, Anemia, Prognosis, Hemoglobin

## Abstract

**Background:**

Anemia is prevalent not only in COPD but also in pulmonary hypertension. We postulated that anemia may have certain prognostic value in COPD concomitant with PH due to COPD (COPD-PH).

**Methods:**

We performed a 12-month prospective investigation to follow up COPD patients with or without PH assessed by right heart catheterization. Eligible patients were enrolled, stratified into COPD-PH-anemia group (*n* = 40), COPD-PH group (*n* = 42), COPD-anemia group (*n* = 48), and COPD group(*n* = 50), and then followed up for 12 months.

**Results:**

After the follow-up, for both of the actual variation value and variation rate, the increase of NT-pro BNP (*P*<0.001; *P* = 0.03) and CAT score (*P* = 0.001; 0.002), as well as the decrease of PaO_2_ (*P* = 0.03; 0.086) and Peak VO_2_ (*P* = 0.021; 0.009) in COPD-PH-anemia group were highest among four groups. The cumulative one-year survival rates were similar among four groups (*P* = 0.434). The cumulative exacerbation-free rate was lowest in COPD-PH-anemia group among four groups (*P*<0.001). Hemoglobin was an independent promoting factor for the probability of hospitalization due to exacerbation ≧ 1/year in patients with COPD-PH-anemia [HR 3.121(2.325–5.981); *P*<0.001].

**Conclusions:**

Anemia is a promoting factor for the worsening of exercise capacity, deterioration of hypoxemia, declining of life quality, and aggravation of exacerbations in patients with COPD-PH-anemia, by contrast with COPD-PH, COPD-anemia, and COPD.

## Background

Chronic obstructive pulmonary disease (COPD) has become the third leading cause of death worldwide and is projected to be the disease with the seventh greatest burden worldwide in 2030. It is a major chronic cause of morbidity and mortality all over the world. Many patients suffer from this disease for many years, and die prematurely due to itself or its complications [1–3].

Pulmonary hypertension (PH) is a pathophysiological disorder involving multiple clinical disciplines, which majorly include multiple cardiovascular and respiratory diseases [4]. It may develop in the advanced stage of COPD and is basically due to hypoxic vasoconstriction of pulmonary capillaries, eventually leading to structural changes which include intimal hyperplasia and the consequent smooth muscle hypertrophy [5–7]. Once PH develops in patients with COPD, what may follow are the deteriorated exercise capacity, worsened hypoxemia and shortened survival [8–10].

In patients with COPD, systemic inflammatory mediators may contribute to skeletal muscular atrophy or cachexia, and initiate or aggravate anemia [11], meanwhile, anemia is common in patients with PH and may be associated with reduced exercise capacity, and with a higher mortality [12–16]. Therefore, since anemia had been evident to be prevalent in both COPD and PH, we wondered how the prognostic role anemia was in PH due to COPD. For patients with COPD related PH, there are many shared prognostic factors between COPD and PH, such as DLcO, 6MWD, PaO_2_, mMRC score and peak VO_2_ which all decline worse than in either of COPD or PH alone providing the inter-related basis for the assessment of COPD-PH. To date, no existing studies have concerned this subject. Therefore, this study was designed to explore the potential prognostic value of anemia in PH due to COPD.

## Methods

### Study design

We performed a 12-month prospective study to investigate the role of anemia in COPD concomitant with PH. All eligible patients were screened out according to inclusion and exclusion criteria and then stratified into COPD-PH-anemia group, COPD-PH group, COPD-anemia group, and COPD group, to be followed up for 12 months. Variables encompassing routine blood test (RBT), COPD assessment test (CAT), pulmonary function test (PFT), cardiopulmonary exercise test (CPET), 6 min walk distance (6MWD), and arterial blood gas analysis (ABGA) were assessed at the baseline and the endpoint. Cumulative exacerbation counting and all-cause mortality were documented during the follow-up. All relevant variables were compared amongst the four groups after the finish of follow-up. During the enrollment of the patients, we equalized the variables of factors which might impact the prognosis to the maximum extent except for hemoglobin. Since our subjects were primarily patients with COPD, so we focused mainly on the equalization of GOLD stage, AE history, and co-morbidities. Meanwhile, we eliminated the difference of therapies by standardizing the treatment according to the guidelines. This protocol was approved by the institutional review board of Shanghai Pulmonary Hospital. Written informed consent was obtained from all patients.

### Study population

All eligible patients were enrolled from a cohort of patients with COPD with or without PH assessed by right heart catheterization (RHC) between 2013 and 2016 of the department of cardiopulmonary circulation of Shanghai Pulmonary Hospital Tongji University, due to at least one of the following reasons: 1) episodes of RV failure or suspected PH by echocardiographic findings; 2) suspected PAH or CTEPH; 3) candidates for lung transplantation or lung volume reduction.

Eligible patients were enrolled according to the inclusion criteria and the exclusion criteria. Inclusion criteria: 1) age ≥ 40 yrs.; 2) a diagnosis of COPD at all stages/groups, defined as an FEV_1_: FVC ratio of less than 0.70 after bronchodilator use plus respiratory symptoms, a history of exposure to risk factors (e.g., smoking, air pollution, biomass combustion), or both, measured 20 min after the inhalation of 400 μg of albuterol (Ventolin, Glaxo Wellcome) [11]; 3) a diagnosis with PH on the presence of mean pulmonary arterial pressure (mPAP) ≧25 mmHg and pulmonary artery wedge pressure (PAWP) ≤18 mmHg in RHC or an exclusion of PH by mPAP< 25 mmHg in RHC [4]; 4) with or without a diagnosis of anemia defined as a hemoglobin concentration of < 13 g/dL for males and 12 g/dL^−^for females [17]. Exclusion criteria: 1) a diagnosis of other chronic pulmonary diseases including, asthma or asthma-COPD overlap (ACO), bronchiectasis, tuberculosis, obliterative bronchiolitis, diffuse panbronchiolitis, interstitial lung disease, or combined pulmonary fibrosis and emphysema; 2) a diagnosis of PH in Group 1, Group 2, Group 4, or Group 5 according to the classifications in 2015 ESC/ERS guidelines [4]; 3) patients with hematological diseases including secondary anemia such as anemia due to cancer or immunological diseases or hemorrhage, or receive regimens which affect hemoglobin except for anti-anemia therapy; 4) patients who lived on plateau all the time; 5) patients who were lost to follow-up or who did not comply with COPD-related or PH-related treatments.

### Assessments

We performed the assessments encompassing several aspects which were exercise capacity, hypoxemia, life quality, acute exacerbation and all-cause mortality. The detailed variables we focused were hemoglobin, carboxyhemoglobin, methemoglobin in RBT, PaO_2_ in ABGA, FEV_1_ of the predicted value in PFT, peak VO_2_ in CPET, CAT score, NT-pro BNP and 6MWD. All assessments were performed when patients were at their stable status. In case of patients happened to be in an exacerbated state at the moment of assessment, the evaluation would be postponed till patients recovered from exacerbations. Exacerbation was defined as an acute worsening of respiratory symptoms that result in additional therapy [18, 19]. At the end of each month during the follow-up, study personnel determined the patients’ status including exacerbations, hospitalizations due to exacerbations, and survival status in the previous month by telephone contact.

### Statistical analysis

According to the prevalence of COPD (11.7%), the anemia prevalence in COPD (12.3–23%), and the prevalence of PH in COPD (50–90%), to ensure the 95% confidential interval, we estimated we at least needed to measure in total of 159 cases of COPD patients, in which at least 82 cases of COPD-anemia in total, 75 cases of COPD-PH in total, and at least 36 cases of COPD-PH-anemia.

Measurement data was presented as mean ± standard deviation or median with interquartile range according to their distribution. Categorical data was presented as frequencies and percentages. Exacerbation-free rates and survival rates at different time-points were estimated by means of Kaplan–Meier method, and any differences between groups were evaluated with a stratified log-rank test. The multiple testing among all groups was conducted by using ANOVA with Bonferroni correction. The change of patients’ variables between the baseline and the study completion was calculated: change = (variable at completion- variable at baseline); the change rate was calculated: change rate = (variable at completion-variable at baseline)/variable at baseline. Cox regression analysis was performed to assess the correlation between variables and the probability of hospitalizations due to exacerbations ≧ 1 time per year. A *p*-value < 0.05 was defined as being of statistical significance.

## Results

### Demographics and characteristics of the patients

This investigation was launched in January, 2016, and finished in December, 2017, following the finish of follow-up of the last enrolled patient. After the exclusion of 10 cases with at least one of the following diagnoses of asthma, bronchiectasis, tuberculosis, obliterative bronchiolitis, diffuse panbronchiolitis, interstitial lung disease, or combined pulmonary fibrosis and emphysema, 6 cases with a diagnosis of PH in Group 1, Group 2, Group 4, or Group 5, and 3 cases with hematological diseases, or receive regimens which affect hemoglobin except for anti-anemia therapy, amongst 207 cases, finally, a total of 188 eligible patients were screened out to access to the follow-up program. Then after the loss of 8 cases to the follow-up, in the end, 180 cases entered into the final full analysis set. Throughout all of them, the cases in COPD-PH-anemia group, COPD-PH group, COPD-anemia group, and COPD group were 40, 42, 48, and 50, respectively. The overall mean age and male/female sex ratio of all eligible patients were 66.1 years and 131/49, respectively. No statistical difference was found among four groups in regard to age, sex ratio, smoking history, AE history, FEV_1_, GOLD stages, and COPD groups (*P* > 0.05 for all comparisons), except for BMI (*P* = 0.025), mPAP (*P* = 0.016), 6MWD (*P* = 0.003), NT-pro BNP (*P*<0.001), PaO_2_ (*P* = 0.006), peak VO_2_(*P* = 0.018) and hemoglobin (*P* = 0.036) at the baseline. By means of a routine blood test, among 88 cases with anemia, 45 cases were identified to be normocytic anemia, 33 cases were microcytic anemia, and 10 cases were macrocytic anemia. Demographics and characteristics of the patients at the baseline were summarized in Table 1. LTOT was prescribed according to patients’ indication before and during the study period. No statistical difference regarding LTOT was found among four groups (*P* = 0.085).

**Table 1:** Demographics and characteristics of all the patients at the baseline

Variables	COPD-PH-anemia (*n* = 40)	COPD-PH (*n* = 42)	COPD-anemia (*n* = 48)	COPD (*n* = 50)	*P* value
Age-yrs	65.9 ± 5.3	68.7 ± 8.1	62.6 ± 6.7	67.2 ± 7.5	0.587
Sex (M/F)-%	75.0/25.0	71.4/28.6	68.8/31.2	76.0/24.0	0.661
BMI-kg/m^2^	18.5 ± 5.8	21.6 ± 6.2	22.4 ± 4.7	25.1 ± 7.1	0.025
Smoking history (Y/N)-%	90.0/10.0	88.1/11.9	85.4/14.6	86.0/14.0	0.549
FEV_1_ of predicted value-%	40.7 ± 25.9	43.8 ± 19.6	47.7 ± 22.6	42.3 ± 17.8	0.383
AE history- no.	2.8 ± 1.5	2.5 ± 2.3	2.6 ± 1.7	2.2 ± 1.8	0.446
GOLD (I/II/III/IV)-%	0/7.5/47.5/45.0	0/11.9/45.2/42.9	0/8.3/41.7/50.0	0/10.0/43.1/46.9	0.125
Group (A/B/C/D)-%	0/12.5/40.0/47.5	0/14.3/47.6/38.1	0/14.6/43.8/41.6	0/16.0/40.0/44.0	0.374
mPAP-mmHg	33.8 ± 18.5	30.2 ± 22.6	20.6 ± 15.3	18.9 ± 25.4	0.016
CAT score-points	23.5 ± 10.2	16.9 ± 13.8	22.2 ± 8.6	18.7 ± 15.1	0.186
6MWD-m	298.9 ± 183.4	353.6 ± 164.7	382.1 ± 173.7	452.3 ± 152.2	0.003
NT-pro BNP-ng/L	1608.2 ± 679.8	1344.9 ± 852.4	556.7 ± 391.1	374.6 ± 421.0	<0.001
PaO_2_-mmHg	45.3 ± 13.8	50.7 ± 18.1	55.6 ± 16.4	65.7 ± 17.2	0.006
Peak VO_2_-ml/min/kg	13.9 ± 5.7	15.2 ± 7.3	18.5 ± 6.2	22.2 ± 8.1	0.018
Hemoglobin-g/dL^−1^	9.4 ± 5.5	14.2 ± 6.7	10.7 ± 8.3	13.8 ± 4.9	0.036

Note: *COPD* chronic obstructive pulmonary disease, *PH* pulmonary hypertension, *BMI* body mass index, *FEV*_1_ forced expiatory volume in 1 s, *AE* acute exacerbation, *GOLD* global Initiative for Chronic Obstructive lung Disease, *mPAP* mean pulmonary arterial pressure, *CAT* COPD assessment test, 6MWD 6-min walking distance, *NT-proBNP* N-terminal pro-brain natriuretic peptide, *PaO*_2_ arterial blood oxygen tension, *Peak VO*_2_ peak oxygen consumption

### Comparison of variation of variables between the baseline and the endpoint among four groups

The results demonstrated that no statistical difference were found regarding the FEV_1_ among four groups in both aspects of actual variation value and variation rate (*P* = 0.057;0.062). Except the variation rates of PaO_2_ were similar among four groups(*P* = 0.086), no matter regarding actual variation value or variation rate, the increase of NT-pro BNP (*P*<0.001;*P* = 0.03) and CAT score (*P* = 0.001;0.002) in COPD-PH-anemia group were significantly highest among four groups, whereas the decrease of PaO_2_ (*P* = 0.03;0.086) and Peak VO_2_ (*P* = 0.021;0.009) in COPD-PH-anemia group were significantly highest among four groups (Table 2).

**Table 2:** Comparison of the change and changing rate of patients’ variables between the baseline and the endpoint among four groups

Variables	COPD-PH-anemia (*n* = 40)	COPD-PH (*n* = 42)	COPD-anemia (*n* = 48)	COPD (*n* = 50)	*P* value
FEV_1_-% (%)	−8.8 ± 3.6(− 10.1 ± 5.3)	−8.5 ± 6.4(− 10.6 ± 4.6)	− 7.2 ± 5.1(− 8.3 ± 7.2)	−7.6 ± 4.2(−9.1 ± 4.8)	0.057(0.062)
CAT score-points (%)	12.6 ± 5.8(23.7 ± 15.1)	6.5 ± 3.7(19.2 ± 12.4)	6.6 ± 4.0(22.3 ± 10.6)	4.7 ± 3.2(20.1 ± 8.8)	0.001(0.002)
6MWD-m (%)	−59.5 ± 45.6(− 32.2 ± 18.8)	−34.3 ± 41.2(− 26.5 ± 14.3)	−28.4 ± 40.1(− 20.5 ± 16.2)	− 19.7 ± 38.3(− 16.5 ± 12.3)	0.007(0.01)
NT-pro BNP- ng/L (%)	597.1 ± 154.4(31.3 ± 20.4)	466.8 ± 191.0(25.5 ± 22.3)	125.7 ± 112.1(19.5 ± 17.2)	133.6 ± 108.5(15.2 ± 13.3)	<0.001(0.03)
PaO_2_-mmHg (%)	−10.7 ± 5.8(− 10.9 ± 8.6)	−7.6 ± 5.3(− 11.7 ± 7.0)	−6.6 ± 5.4(−8.8 ± 7.5)	− 4.9 ± 4.5(− 7.9 ± 7.2)	0.03(0.086)
Peak VO_2_-ml/min/kg (%)	−3.5 ± 1.6(− 32.4 ± 10.3)	−2.8 ± 1.9(− 25.5 ± 13.6)	−2.4 ± 2.1(− 17.7 ± 8.8)	−1.8 ± 1.4(− 10.3 ± 11.7)	0.021(0.009)

Note: *FEV*_1_ forced expiatory volume in 1 s, *CAT* COPD assessment test, 6*MWD* 6-min walking distance, *NT-proBNP* N-terminal pro-brain natriuretic peptide, *PaO*_2_ arterial blood oxygen tension, *Peak VO*_2_ peak oxygen consumption

### Comparison of cumulative overall survival, exacerbation-free rate amongst four groups

**Fig. 1:**
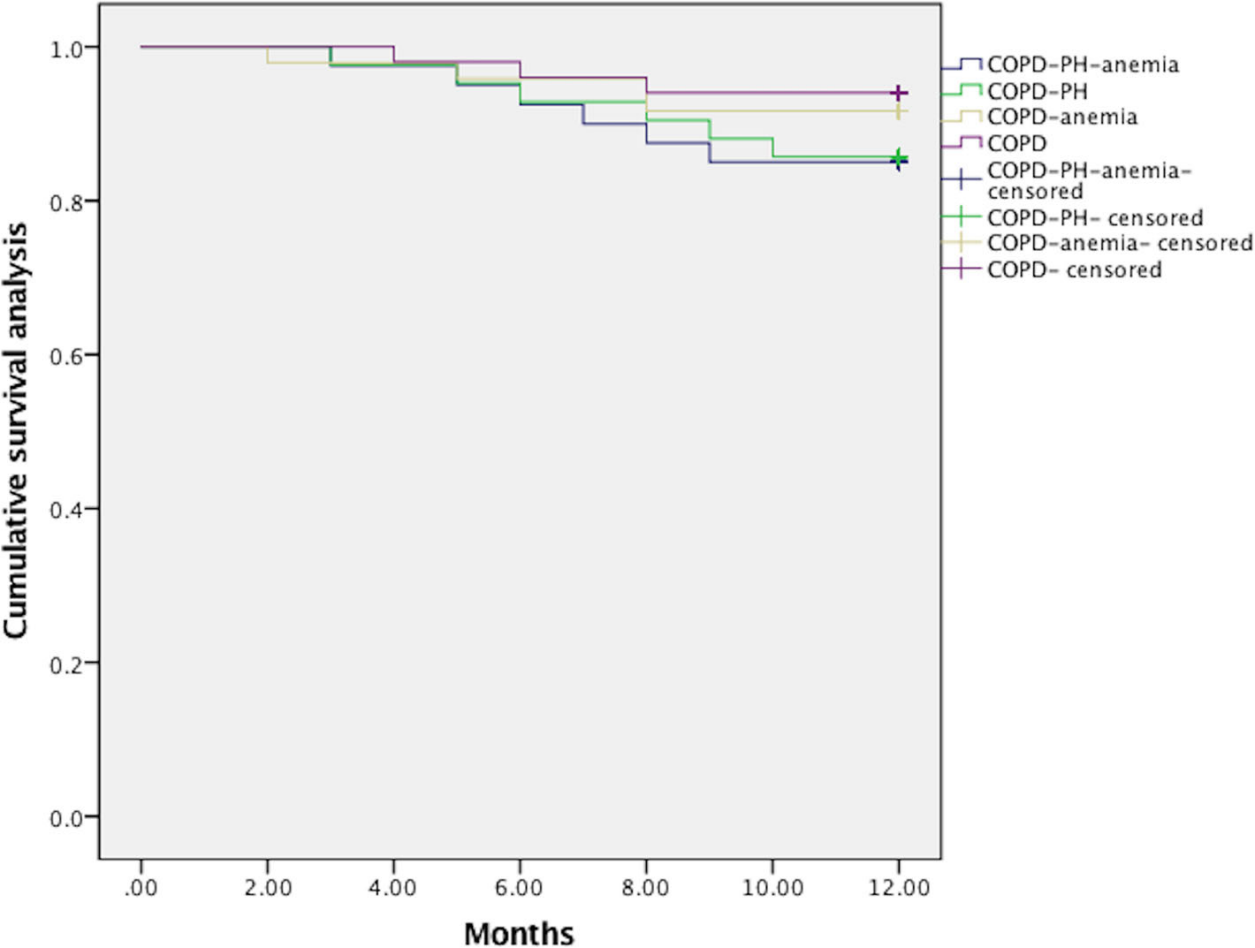
Comparison of cumulative one-year overall survival rate among COPD-PH-anemia group, COPD-PH group, COPD-anemia group, and COPD group (*P* = 0.434)

**Fig. 2:**
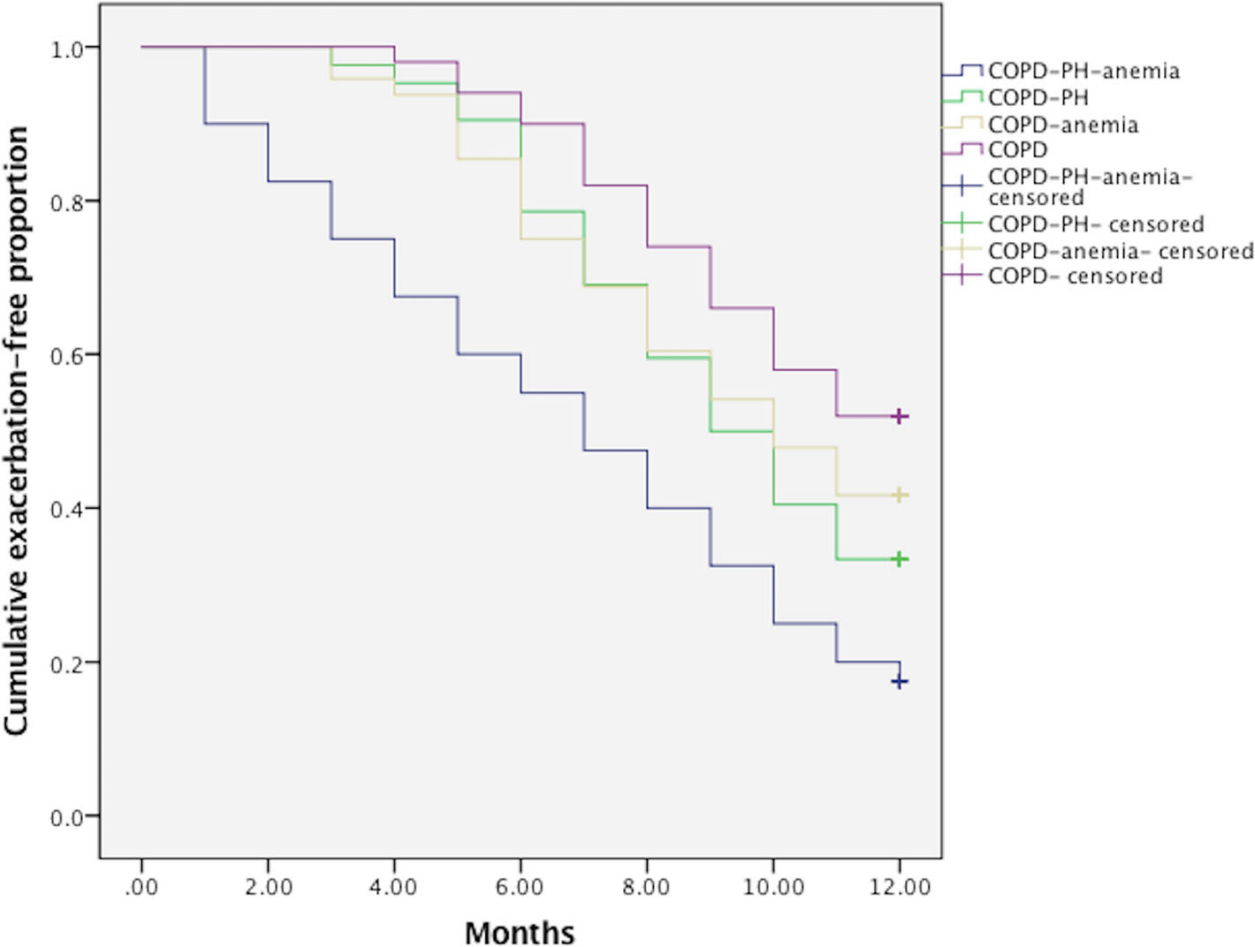
Comparison of cumulative one-year exacerbation-free rate among COPD-PH-anemia group, COPD-PH group, COPD-anemia group, and COPD group (*P*<0.001)

At the end of the follow-up, the cumulative overall mortality were 19 cases, in which 7cases were in COPD-PH-anemia group, 5 cases were in COPD-PH group, 4 cases were in COPD-anemia group, and 3cases were in COPD group(*P* = 0.096). Among all the deceased, 10 patients died of respiratory failure, 7 patients died of heart failure, 2 cases died of sudden death. In a Kaplan–Meier analysis, the results demonstrated that the cumulative one-year survival rates were similar amongst COPD-PH-anemia group, COPD-PH group, COPD-anemia group, and COPD group (*P* = 0.434) (Fig. 1.) Throughout the whole process of follow-up, the mean annual exacerbations or hospitalizations counting per patient were 3.5 and 1.8 times in COPD-PH-anemia group, 2.6 and 1.7 times in COPD-PH group, 2.4 and 1.3 times in COPD-anemia group, as well as 1.8 and 0.8 times in COPD group, respectively (*P* = 0.005;*P* = 0.018). At the end of the follow-up, the cases with at least one exacerbation or one hospitalization were 118(65.6%) and 66 (36.7%) cases, respectively. The prevalence of exacerbations or hospitalizations were 35(87.5%) and 16(40.0%) in COPD-PH-anemia group, 28(66.7%) and 15(35.7%) in COPD-PH group, 30(62.5%) and 12(25.0%) in COPD-anemia group, as well as 25 (50%) and 10(20.0%) in COPD group [*P* = 0.033(*P*<0.001);*P* = 0.065(*P* = 0.005)]. In a Kaplan–Meier analysis, the results demonstrated that the cumulative exacerbation-free proportion was lowest in COPD-PH-anemia group, and highest in COPD group, whereas no statistical difference was found between COPD-PH group and COPD-anemia group(*P*<0.001) (Fig. 2.).

### Correlation between risk factors and exacerbations in each group by multivariate regression analysis

After an univariate analysis between risk factors and the development of hospitalizations due to exacerbations ≧ 1/year, then adjusting for age, sex, smoking history and BMI, a multivariate analysis demonstrated that, for patients with COPD-PH-anemia, along with per decrease of 1 g/dL^− 1^ of hemoglobin, the hazard ratio of hospitalizations ≧ 1/year was 3.121, being similar with some variables such as AE history and COPD groups. Also in a multivariate regression analysis between dyshemoglobins which were carboxyhemoglobin as well as methemoglobin and the risk for hospitalizations ≧ 1/year, the results showed that only carboxyhemoglobin was positively correlated with the development of hospitalizations ≧ 1/year especially in COPD-PH-anemia group (Table 3.)

**Table 3:** Correlation between risk factors and the probability of hospitalizations ≧ 1/year in each group by multivariate regression analysis

Variables	COPD-PH-anemia (*n* = 40)	COPD-PH (*n* = 42)	COPD-anemia (*n* = 48)	COPD (*n* = 50)
	HR (95%CI), *P* value	HR (95%CI), *P* value	HR (95%CI), *P* value	HR (95%CI), *P* value
FEV_1_-per decrease of 10%	2.565(1.225–3.764) 0.003	2.439(1.219–3.664) 0.002	2.108(1.321–4.214) 0.005	1.884(0.871–3.265) 0.003
AE history-per increase of 1 time	3.338(1.532–4.698) <0.001	3.157(1.339–5.310)< 0.001	2.541(1.210–4.311)0.001	2.226(1.722–3.827)0.001
GOLD-per progression of 1 stage	2.765(1.555–3.827)0.008	2.672(1.246–3.981)0.005	2.519(1.433–3.646)0.001	1.987(0.592–3.218)0.037
Group-per progression of 1 level	3.102(1.426–3.223) <0.001	2.453(1.002–3.528)0.002	2.313(1.038–3.297)0.004	2.124(0.762–3.281)0.014
PaO_2_-per decrease of 10 mmHg	2.384(1.542–3.456)0.02	2.176(1.256–3.642)0.006	2.987(1.777–3.562)0.007	1.733(0.888–3.213)0.028
Peak VO_2_-per decrease of 10 ml/min/kg	2.182(1.214–3.628)0.003	2.815(1.118–3.823)0.033	2.143(1.143–3.427)0.004	2.054(1.076–3.665)0.029
6MWD-per decrease of 100 m	1.676(0.662–3.186)0.036	1.501(0.855–3.222)0.044	1.453(0.337–3.212)0.038	1.222(0.443–3.251)0.057
Hemoglobin -per decrease of 1 g/dL^− 1^	3.121(2.325–5.981) <0.001	1(reference)	2.756(1.985–3.784) <0.001	1(reference)
Carboxyhemoglobin-per increase of 0.1 g/dL^−1^	2.838(1.698–5.210)0.001	2.663(1.520–3.228)0.001	2.437(1.265–3.884)0.001	1.688(1.104–3.651)0.002

Note: *COPD* chronic obstructive pulmonary disease, *PH* pulmonary hypertension, *FEV*_1_ forced expiatory volume in 1 s, *AE* acute exacerbation, *GOLD* global Initiative for Chronic Obstructive lung Disease, *PaO*_2_ arterial blood oxygen tension, *Peak VO*_2_ peak oxygen consumption, 6*MWD* 6-min walking distance

## Discussion

In consideration of anemia may have certain prognostic value in patients with pulmonary hypertension due to COPD, whereas little of them was known quoad hoc, thus we performed this study. In this study, we found that, among COPD-PH-anemia group, COPD-PH group, COPD-anemia group, and COPD group, the patients in COPD-PH-anemia group had the most deterioration in exercise capacity, hypoxemia, life quality, and highest risk of acute exacerbations, except for the similar overall survival rates among all groups, in a 12-month interval.

To our best knowledge, no existing comparable study is eligible to be the contrast with this study, therefore, what we can discuss hereby is this investigation exclusively. Since PH is also a concomitant co-morbidity just like anemia, we primarily regarded the subjects as COPD patients, then as PH or not. In order to present the the impact of anemia on COPD-PH to the maximum extent, we set up not only COPD-PH, but also COPD-anemia and COPD as control. Besides the information of impact of anemia on COPD-PH, we could also obtain the information regarding the different impact of anemia on COPD-PH and COPD, respectively, by contrast with sole COPD. It cannot be denied that secondary polycythemia is a common phenomenon in patients with COPD just like anemia, in other words, the two pathophysiologic processes may potentially happen in patients with COPD simultaneously, especially in early stage of COPD. Therefore, since the basic hemoglobin level of COPD may be higher than that of normal person, we adopted the diagnostic criteria of WHO for anemia which are < 13 g/dL for males and < 12 g/dL for females, respectively, instead of the criteria of anemia in China which are <12 g/l for male and <11 g/l for female, respectively, to eliminate the potential confounding of secondary polycythemia.

To start with, except for hemoglobin which was predetermined to be different among four groups, the demographics showed that no statistical difference was found in regard to age, sex ratio, smoking history, AE history, FEV_1_, GOLD stages, and COPD groups, suggesting the homogeneity was considerable at least from the perspective of COPD, among all eligible patients at the baseline. Nevertheless, some variables such as mPAP, 6MWD, NT-pro BNP, PaO_2_, and peak VO_2_ were heterogenous among all eligible patients at the baseline partially attributable to the role of PH. Interestingly, the BMI in COPD-PH-anemia group was lowest among four groups suggesting anemia may interrelate with nutritional status. It is noteworthy that the cause of anemia was majorly due to normocytic type which conformed to the characteristics of COPD [11]. As for microcytic type being the second major cause, we believe it is related to PH [12–16].

After the follow-up, the results showed no dramatic variation regarding the FEV_1_ which is a COPD-related variable concerning airflow limitation, whereas the variations of NT-pro BNP, CAT score, PaO_2_ and Peak VO_2_ were significant among four groups in which the COPD-PH-anemia group had the worst deterioration. This indicated that, anemia impacted more seriously on patients with COPD-PH than on mere COPD, encompassing the perspectives of life quality, ventricular dysfunction, and hypoxemia especially whilst exercise, except for airflow limitation. On account of the impairment of oxygen-transporting function in anemia, patients with COPD-PH-anemia are naturally more liable to develop ingravescent fatigue, heart failure and hypoxemia rather than airflow limitation, by contrast with either COPD-PH or COPD.

The next comparison of cumulative overall survival showed no difference of cumulative one-year survival rates among four groups. This could be interpreted as that anemia makes no difference on the survival of patients with COPD-PH or COPD for at least 1 year. By contrast, in the study of Pernille et al., anemia could be used to predict mortality. In view of Pernille’s study was a five-year retrospective review, while ours was a one-year prospective investigation, the investigating period in this study may be too limited to uncover the difference of mortality among different groups [20].

In the study of Pernille, et al., low level of hemoglobin are frequent in COPD patients with acute exacerbations [20]. In our study, the comparison of exacerbations demonstrated that COPD-PH-anemia group had the most mean annual exacerbations or hospitalizations counting, the highest prevalent rate of exacerbations or hospitalizations, and lowest cumulative exacerbation-free rate among patients among four groups. It means that, by contrast with simple COPD, anemic COPD, or simple COPD-PH, COPD-PH-anemia has the highest risk for developing an exacerbation. It is believed that, by deteriorating life quality, ventricular dysfunction, and hypoxemia, anemia contributes to the aggravation of exacerbations.

The last correlation analysis between risk factors and hospitalizations showed that, being similar with some exacerbation-related classical predictors in COPD such as AE history and COPD groups [11], hemoglobin was an independently contributing factor for the probability of hospitalizations ≧ 1/year in COPD patients especially patients with COPD-PH-anemia. Decremental hemoglobin is a promoting factor for the incremental exacerbations or hospitalizations. By the way, we also performed a correlation analysis between some dyshemoglobins which were carboxyhemoglobin as well as methemoglobin and hospitalizations. The results demonstrated that carboxyhemoglobin was positively correlated with the development of hospitalizations ≧ 1/year in all four groups especially in COPD-PH-anemia group rather than methemoglobin. Likewise, in the study of Yasuda et al., the carboxyhemoglobin level at exacerbations were significantly higher than those at stable stage, the increased arterial carboxyhemoglobin was correlated to the severity of COPD resulting from systemic inflammation and reactive oxygen species [21].

Some systematic inflammatory diseases such as connective tissue disease are frequently concomitant with anemia of chronic disease through the mechanism of the production of inflammatory mediators damaging the generation of erythrocytes. Likewise, COPD which is one of systematic inflammatory diseases is generally concomitant with the elevation of IL-1, IL-6 and TNF-a level in circulation inducing the development of anemia [22]. Some studies demonstrated that anemia was closely related to C reactive protein which is an inflammatory biomarker [23, 24]. Besides, inflammatory mediators may also result in skeletal muscular atrophy and cachexia further deteriorating anemia [11]. On the other hand, patients with pulmonary hypertension commonly develop right ventricular dysfunction in which 15% are concomitant with anemia [25–28]. Its mechanism is due to the release of inflammatory mediators whilst heart failure, the activation of renin-angiotensin system [28]. All these may explain the impressive prevalence of anemia in COPD-PH.

The clinical implications of this study are considered to be the following: first, the results of our study may urge clinicians to be aware of the serious prevalence of anemia in COPD patients concomitant with PH; second, clinician could be vigilant about the severely adverse impact of anemia on the prognosis of COPD-PH in order to inform patients’ family members timely and take action in advance; third, under some circumstances in which a dilemma exists in the assessment of prognosis, anemia could be an eligible weight which can be taken into account.

The strength of this study consisted in: first, the eligible patients being studied all underwent RHC which is the only gold standard for the diagnosis of PH to date, to ascertain wether they had PH or not, ensuring the eligibility of PH-negative COPD controls; second, we compared the longitudinal variation and variation rate between the baseline and the endpoint instead of comparing the variables at the endpoint, to reflect the time-dependent impact that anemia would result in. Nevertheless, several limitations existed in this study. First, the sample size was not very large due to the nature of prospective investigation. A large-scale study is warranted in the future. Second, obviously we have no comments to make on the potential difference of overall survival amongst different groups beyond one-year follow-up which might be too short to show the discrepancy. The last but not least, in view of the patients being reviewed in this study were all Chinese patients, the results of this study may not be applicable for other races.

## Conclusions

In summary, in this study, we may draw a conclusion that anemia is a promoting factor for worse deterioration of exercise capacity, deterioration of hypoxemia, declining of life quality, as well as aggravation of exacerbations or hospitalizations in patients with COPD-PH-anemia, by contrast with patients with COPD-PH, COPD-anemia, or COPD.

## Abbreviations

6MWD: 6 min walk distance
ABGA: Arterial blood gas analysis
AE: Acute exacerbation
BMI: Body mass index
CAT: COPD assessment test
COPD: Chronic obstructive pulmonary disease
CPET: Cardiopulmonary exercise test
CTEPH: Chronic thromboembolic pulmonary hypertension
FEV_1_: Forced expiatory volume in 1 s
GOLD: Global Initiative for Chronic Obstructive lung Disease
mPAP: Mean pulmonary arterial pressure
NT-proBNP: N-terminal pro-brain natriuretic peptide
PAH: Pulmonary arterial hypertension
PaO_2_: Arterial blood oxygen tension
PAWP: Pulmonary artery wedge pressure
Peak VO_2_: Peak oxygen consumption
PFT: Pulmonary function test,
PH: Pulmonary hypertension
RBT: Routine blood test
RHC: Right heart catheterization
RV: Right ventricular

## References

[1] Lozano R, Naghavi M, Foreman K (2012). Global and regional mortality from 235 causes of death for 20 age groups in 1990 and 2010: a systematic analysis for the global burden of disease study 2010. Lancet.

[2] Yang G, Wang Y, Zeng Y (2013). Rapid health transition in China, 1990-2010: findings from the global burden of disease study 2010. Lancet.

[3] Mathers CD, Loncar D (2006). Projections of global mortality and burden of disease from 2002 to 2030. PLoS Med.

[4] Galiè N, Humbert M, Vachiery J-L (2015). 2015 ESC/ERS guidelines for the diagnosis and treatment of pulmonary hypertension. Eur Respir J.

[5] Sakao S, Voelkel NF, Tatsumi K (2014). The vascular bed in COPD: pulmonary hypertension and pulmonary vascular alterations. Eur Respir Rev.

[6] Peinado VI, Pizarro S, Barbera JA (2008). Pulmonary vascular involvement in COPD. Chest.

[7] Wells JM, Washko GR, Han MK (2012). Pulmonary arterial enlargement and acute exacerbations of COPD. N Engl J Med.

[8] Oswald-Mammosser M, Weitzenblum E, Quoix E (1995). Prognostic factors in COPD patients receiving long-term oxygen therapy. Importance of pulmonary artery pressure. Chest.

[9] Kessler R, Faller M, Weitzenblum E (2001). “Natural history” of pulmonary hypertension in a series of 131 patients with chronic obstructive lung disease. Am J Respir Crit Care Med.

[10] Lettieri CJ, Nathan SD, Barnett SD (2006). Prevalence and outcomes of pulmonary arterial hypertension in advanced idiopathic pulmonary fibrosis. Chest.

[11] Global Strategy for the Diagnosis, Management and Prevention of COPD, Global Initiative for Chronic Obstructive Lung Disease (GOLD). Publication list; 2017. http://goldcopd.org/gold-2017-global-strategy-diagnosis-management-prevention-copd/.

[12] Ruiter G, Lankhorst S, Boonstra A (2011). Iron deficiency is common in idiopathic pulmonary arterial hypertension. Eur Respir J.

[13] Ruiter G, Lanser IJ, de Man FS (2014). Iron deficiency in systemic sclerosis patients with and without pulmonary hypertension. Rheumatology (Oxford).

[14] Broberg CS, Bax BE, Okonko DO (2006). Blood viscosity and its relationship to iron deficiency, symptoms, and exercise capacity in adults with cyanotic congenital heart disease. J Am Coll Cardiol.

[15] Rhodes CJ, Howard LS, Busbridge M (2011). Iron deficiency and raised hepcidin in idiopathic pulmonary arterial hypertension clinical prevalence, outcomes, and mechanistic insights. J Am Coll Cardiol.

[16] Van De Bruaene A, Delcroix M, Pasquet A (2011). Iron deficiency is associated with adverse outcome in Eisenmenger patients. Eur Heart J.

[17] World Health Organization (2001). Iron deficiency anemia. assessment, prevention, and control. A guide for programme managers.

[18] Wedzicha JA, Seemungal TA (2007). COPD exacerbations: defining their cause and prevention. Lancet.

[19] Seemungal TA, Donaldson GC, Paul EA (1998). Effect of exacerbation on quality of life in patients with chronic obstructive pulmonary disease. Am J Respir Crit Care Med.

[20] Pernille A, Petersen T, Pedersen CT (2016). Association between hemoglobin and prognosis in patients admitted to hospital for COPD. Int J COPD.

[21] Yasuda H, Yamaya M, Nakayama K (2005). Increased arterial carboxyhemoglobin concentrations in chronic obstructive pulmonary disease. Am J Respir Crit Care.

[22] Weiss G, Goodnough LT (2005). Anemia of chronic disease. N Engl J Med.

[23] John M, Hoernig S, Doehner W (2005). Anemia and inflammation in COPD. Chest.

[24] Markoulaki D, Kostikas K, Papatheodorou G (2011). Hemoglobin, erythropoietin and systemic inflammation in exacerbations of chronic obstructive pulmonary disease. Eur J Intern Med.

[25] Tanner H, Moschovitis G, Kuster GM (2002). The prevalence of anemia in chronic heart failure. Int J Cardiol.

[26] Cromie N, Lee C, Struthers AD (2002). Anaemia in chronic heart failure: what is its frequency in the UK and its underlying causes?. Heart.

[27] Ezekowitz JA, McAlister FA, Armstrong PW (2003). Anemia is common in heart failure and is associated with poor outcomes: insights from a cohort of 12065 patients with new-onset heart failure. Circulation.

[28] Okonko DO, Anker SD (2004). Anemia in chronic heart failure: pathogenetic mechanisms. J Card Fail.

